# Photodynamic Therapy Using Systemic Administration of 5-Aminolevulinic Acid and a 410-nm Wavelength Light-Emitting Diode for Methicillin-Resistant *Staphylococcus aureus*-Infected Ulcers in Mice

**DOI:** 10.1371/journal.pone.0105173

**Published:** 2014-08-20

**Authors:** Kuniyuki Morimoto, Toshiyuki Ozawa, Kunio Awazu, Nobuhisa Ito, Norihiro Honda, Sohkichi Matsumoto, Daisuke Tsuruta

**Affiliations:** 1 Department of Dermatology, Osaka City University Graduate School of Medicine, Osaka, Japan; 2 Medical Beam Physics Laboratory, Osaka University Graduate School of Engineering, Osaka, Japan; 3 Department of Bacteriology, Osaka City University Graduate School of Medicine, Osaka, Japan; MGH, MMS, United States of America

## Abstract

Bacterial resistance to antibiotics has become a worldwide problem. One potential alternative for bacterial control is photodynamic therapy. 5-aminolevulinic acid is a natural precursor of the photosensitizer protoporphyrin IX. Relatively little is known about the antibacterial efficacy of photodynamic therapy using the systemic administration of 5-aminolevulinic acid; a few reports have shown that 5-aminolevulinic acid exerts photodynamic effects on methicillin-resistant *Staphylococcus aureus* (MRSA) *in* *vitro*. In this study, we evaluated the effectiveness of photodynamic therapy using 5-aminolevulinic acid and a 410-nm wavelength light-emitting diode *in* *vitro* and *in* *vivo* for the treatment of MRSA. We found that 5-aminolevulinic acid photodynamic therapy with the light-emitting diode had an *in-vitro* bactericidal effect on MRSA. *In vivo*, protoporphyrin IX successfully accumulated in MRSA on ulcer surfaces after intraperitoneal administration of 5-aminolevulinic acid to mice. Furthermore, 5-aminolevulinic acid photodynamic therapy accelerated wound healing and decreased bacterial counts on ulcer surfaces; in contrast, vancomycin treatment did not accelerate wound healing. Our findings indicate that 5-aminolevulinic acid photodynamic therapy may be a new treatment option for MRSA-infected wounds.

## Introduction

*Staphylococcus aureus* is responsible for severe infections in the hospital and the com*m*unity. Due to the spread of methicillin-resistant *S. aureus* (MRSA), glycopeptide antibiotics such as vancomycin (VCM) and teicoplanin are used to treat severe staphylococcal infections [1]. However, extensive use of these antibiotics has led to the appearance of VCM- and methicillin-resistant *S. aureus* [2], [3], necessitating alternative approaches against MRSA toward which no resistance can develop.

One of the most promising and innovative approaches in this respect is antimicrobial photodynamic therapy (PDT) [4], [5]. PDT involves the selective photosensitization of target cells using topically or systemically administered agents that can be activated by light to produce an oxygen-dependent cytotoxic reaction [6], [7]. Methodologically, PDT is performed with 5-aminolevulinic acid (5-ALA), a natural amino acid that is the precursor of a strong photosensitizer, protoporphyrin IX (PpIX), within cells [8]. 5-ALA is water soluble and can be administered locally, systemically, and orally [9], [10]. PpIX is maximally activated at 410 nm in the Soret Band of visible light, with significant lesser peaks of activation at 509, 544, 584, and 635 nm [11]. Until recently, clinically approved indications for PDT were restricted to actinic keratosis [12], [13], nodular and superficial basal cell carcinoma [14], [15], and Bowen’s disease [13], [16], [17]. However, the range of indications for PDT has expanded to the treatment of Gram-positive and Gram-negative bacteria, fungi, viruses, and parasites [18], [19].

Since the probability of the appearance of PDT-resistant bacteria is low, it may serve as a new treatment for bacterial infection [5], [20]. However, relatively little is known about the mechanisms underlying the antibacterial efficacy of 5-ALA-mediated PDT (ALA-PDT) on skin infection by MRSA. 5-ALA can be administered systemically and has been successfully used to diagnose and treat neoplastic disease. ALA-PDT could be used to treat extensive wound infection with MRSA, if 5-ALA in the exudate is taken up by MRSA on the wound surface and if PpIX accumulates in MRSA after the systemic administration of 5-ALA.

The purpose of the present study was to evaluate the effectiveness of ALA-PDT via systemic administration of 5-ALA and a 410-nm wavelength light-emitting diode (LED) on experimental MRSA-infected cutaneous ulcers on the backs of mice. This investigation is the first in which ALA-PDT is carried out on MRSA-infected skin using whole-body administration of 5-ALA.

## Materials and Methods

### Bacterial strain and growth conditions

MRSA was obtained from the American Type Culture Collection (33591) for use in these experiments. Bacteria were routinely grown overnight in tryptic soy broth (Difco, Detroit, MI, USA) under aerobic conditions at 37°C.

### Photosensitizer and light source

5-ALA (SBI Pharmaceuticals Co., Ltd., Tokyo, Japan) was diluted in phosphate-buffered saline just prior to use. Light for photoactivation was generated with an LED (Ushio Inc., Tokyo, Japan) with a major wavelength of 410 nm. The fluence of delivered light was 164.5 mW/cm^2^ at a distance of 4.27 cm.

### *In vitro* ALA-PDT for MRSA using a 410-nm LED

Aliquots (500 µL) of an MRSA suspension (∼10^8^ colony forming units (CFUs)/mL) were transferred to separate wells of a 12-well plate. Equal volumes of 5-ALA were added, resulting in final concentrations of 0.5–25 mg/mL. After 4 h in the dark, the wells were exposed to the LED at 5, 20, and 50 J/cm^2^. For each well, 10-fold serial dilutions were made and cultured on tryptic soy agar at 37°C for 24 h. CFUs were counted visually, and results were expressed as mean values ± standard errors of the mean (SEMs; *N* = 5).

### Cytotoxicity of ALA-PDT using a 410-nm LED

The cytotoxicity of ALA-PDT with a 410-nm LED was determined by performing the colony-forming assay with mouse embryo NIH/3T3 fibroblasts. These cells were purchased from DS Pharma Biomedical (Osaka, Japan) and grown in 35-mm Petri dishes containing Dulbecco’s Modified Eagle Medium (Invitrogen, Carlsbad, CA, USA) supplemented with 10% fetal bovine serum (Invitrogen) and 50 U/mL penicillin at 37°C under a humidified atmosphere with 5% CO_2_. After 18–24 h, 5-ALA was added to the medium at a final concentration of 5 mg/mL. Cells were cultured for another 4 h, then irradiated with the LED at 5, 20, or 50 J/cm^2^. After irradiation, the medium was removed and the cells were detached using 2 mL trypsin-0.25% ethylenediaminetetraacetic acid. Subsequently, the cells were added to 150-mm Petri dishes with 50 mL of fresh culture medium and incubated at 37°C for 10 days, after which cell viability was assessed by staining colonies with crystal violet. Three culture dishes were analyzed in each group.

### Mouse model of MRSA-infected cutaneous ulcers

For *in-vivo* experiments, 8 to 10-week-old male C57BL/ksj *db/db* mice (Japan SLC, Hamamatsu, Japan) were used. The animals were housed at one mouse per cage and maintained on a 12-h light/dark cycle with access to food and water *ad libitum*. All animal procedures were approved by the Laboratory Animal Center of Osaka City University Graduate School of Medicine (Permit Number: 11015). The mice were individually anesthetized via intraperitoneal injection of sodium pentobarbital solution (35 mg/kg). The back was shaved with an electric razor, followed by a depilatory agent.

**Figure 1:**
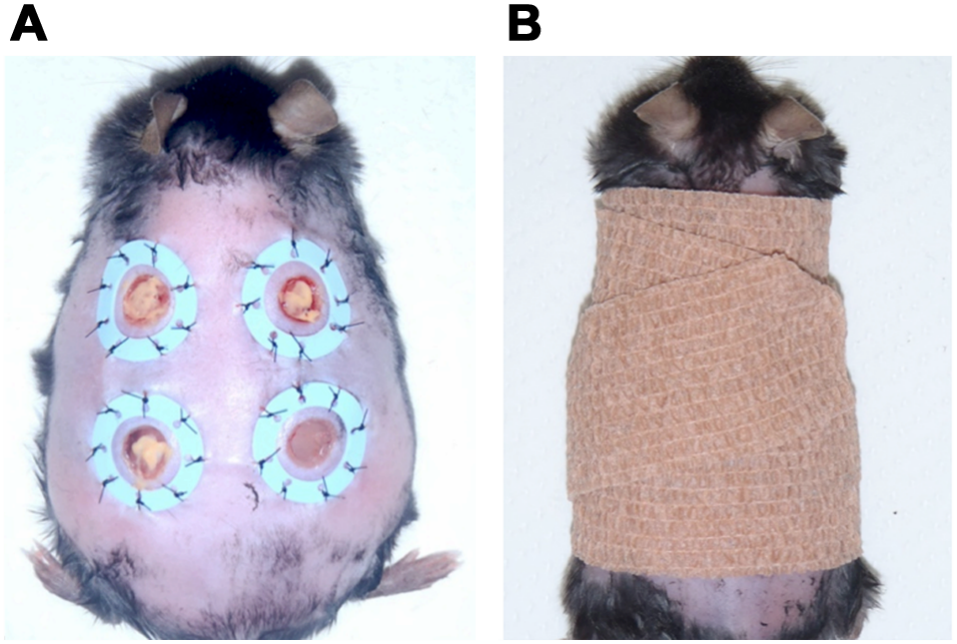
Mouse model of MRSA-infected cutaneous ulcers. (A) Mouse model of infected cutaneous ulcers after inoculation with MRSA. (B) Light shielding and wound protection with a bandage.

On the back of each mouse, a 6-mm punch biopsy tool was used to create full-thickness skin defects 6 mm in diameter that extended through the panniculus carnosus. Four ulcers were made on each mouse, with two ulcers on each side of the midline. A circular silicone splint (inner diameter 10 mm, outer diameter 16 mm) was placed so that the ulcer was centered within the splint. Instant adhesive (Aron Alpha A, Sankyo, Tokyo) was used to fix the splint, and interrupted 5-0 nylon sutures (Ethicon, Inc., Somerville, NJ, USA) were placed to maintain the splint’s position [21] (Figure 1A). The splint was used to minimize wound contracture, allowing healing to occur via epithelialization and the formation of granulation tissue [21]. The ulcers were inoculated with MRSA (10^10^ CFU/cm^2^) and an occlusive dressing (Tegaderm, 3M Health Care, USA) was placed to cover the ulcers. Next, a bandage (Corban, 3M Health Care) was wrapped around each animal to protect the shaved skin and the ulcers (Figure 1B). This state was maintained for 2 days to prepare the mouse model of MRSA-infected cutaneous ulcers.

### PpIX distribution within smears on cutaneous ulcers infected with MRSA

One day after infection, 5-ALA was freshly dissolved in 0.5 mL of saline and injected intraperitoneally at a dose of 200 mg/kg. On day 2, the PpIX distribution within the smear on each MRSA-infected cutaneous ulcer was examined via confocal laser scanning microscopy (TCS SP5, Leica Microsystems). Optical excitation was achieved with a 405-nm diode laser beam, and fluorescence emission was detected at 620–650 nm.

### *In-vivo* ALA-PDT and changes in ulcer area over time

Day 1 was defined as the first day of PDT (two days after cutaneous ulcers were created and inoculated with MRSA). On the day after ulcer creation, 5-ALA was administered systemically via intraperitoneal injection. PDT was performed every day from day 1, and digital photographs were taken before PDT on a daily basis thereafter. 5-ALA was administered intraperitoneally every day immediately after PDT. After irradiation, a bandage was wrapped around each animal to protect the shaved skin and the ulcers. The size of the wound area was measured by tracing the wound margin and calculating the area in pixels using ImageJ 1.46r (National Institutes of Health). The ulcer area on day 1 was defined as 100%, and the ratio of ulcer area after each day of PDT to that on day 1 was calculated. The ratio became 0% upon completion of epithelialization. Five animals were analyzed in each group.

### Subcutaneous antibiotic therapy

Based on a previous report [22], mice were given subcutaneous injections of VCM at a therapeutic dosage (110 mg/kg twice daily). Five animals were analyzed.

### Bacterial counts in wound tissue

Seven days after PDT or treatment with VCM, part of the ulcer (5 mm in diameter) was excised aseptically to the level of the deep fascia using 5-mm punch biopsy forceps. The ulcer samples were homogenized in 1 mL of saline. Viable bacteria in the saline solution were counted by making 10-fold serial dilutions and culturing the dilutions on tryptic soy agar for 24 h at 37°C, after which CFUs were counted manually. Three animals were tested in each group.

### Statistical analysis

All data are presented as mean ± SEM. The significance of differences between sample means was determined by Student’s *t*-test using GraphPad Prism 4.0 (GraphPad, San Diego, CA, USA).

## Results

### Effect of ALA-PDT on MRSA *in* *vitro*

**Figure 2:**
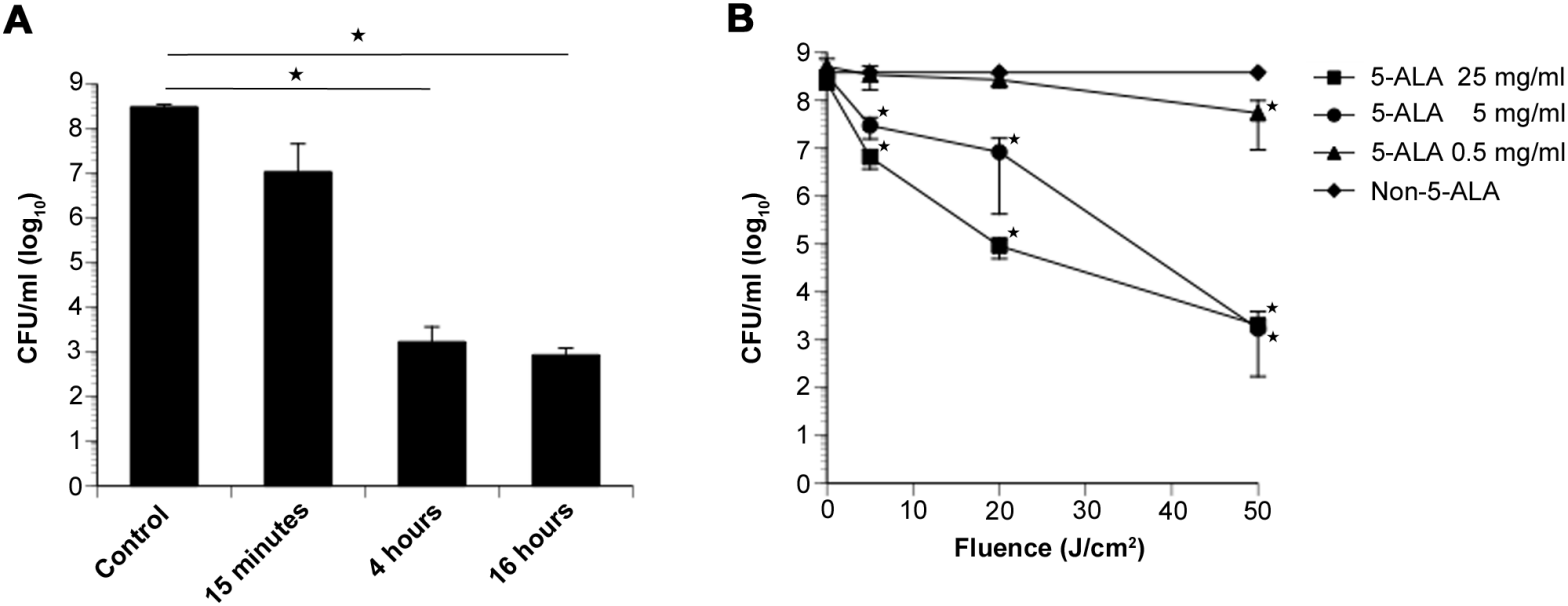
MRSA counts after PDT with 5-ALA *in* *vitro*. (A) MRSA counts (in CFU/mL) 15 min, 4 h, and 16 h after ALA-PDT with 5 mg/mL 5-ALA and LED irradiation at 50 J/cm^2^. (B) MRSA counts after ALA-PDT with various 5-ALA concentrations and LED total fluencies. Data are expressed as mean ± SEM. *P<0.01.

We investigated the antibacterial effect of ALA-PDT on MRSA using a 410-nm LED. CFUs were counted after PDT with various 5-ALA exposure times, 5-ALA concentrations, and total flux levels. Exposure to 5 mg/mL 5-ALA for 15 min before PDT at 50 J/cm^2^ decreased the number of CFUs by 1.3 log_10_-units, whereas 4 h and 16 h of exposure before PDT resulted in a decrease of 5 log_10_-units (Figure 2A). The antibacterial effect of ALA-PDT was dependent on the 5-ALA concentration (0, 0.5, 5, or 25 mg/mL) and the irradiation dose (5, 20, or 50 J/cm^2^) when 5-ALA exposure was carried out for 4 h (Figure 2B). LED irradiation without 5-ALA led to no change in CFU even after increasing the irradiation dose, and exposure to 0.5 mg/mL 5-ALA induced few changes in CFU even after increasing the irradiation dose. With exposure to 5 or 25 mg/mL 5-ALA, decreasing CFUs were observed with increasing LED irradiation doses. When the irradiation dose was 50 J/cm^2^, a similar decrease of 5 log_10_-units was observed at both of these 5-ALA concentrations. These results indicate that ALA-PDT with a 410-nm LED exerts an *in-vitro* antibacterial effect on MRSA.

### ALA-PDT selectivity of MRSA over 3T3 cells

**Figure 3:**
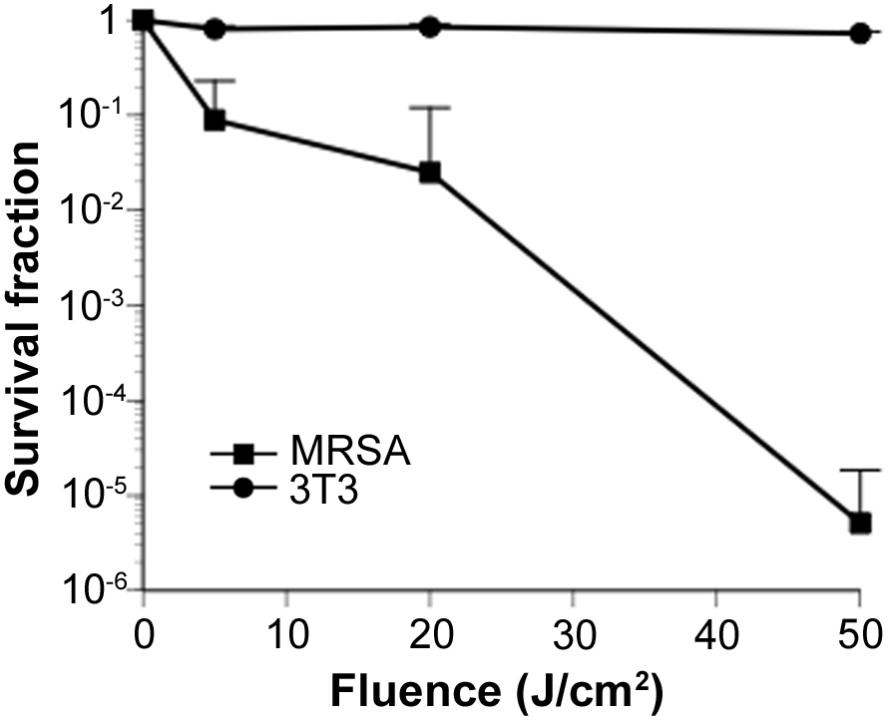
ALA-PDT selectivity of MRSA over 3T3 cells. *In-vitro* survival curves of MRSA and 3T3 cells after PDT with 5 mg/mL 5-ALA and irradiation at 0, 5, 20, and 50 J/cm^2^ with a 410-nm LED. Data are expressed as mean ± SEM. *N* = 3 in each group.

We investigated the selectivity of ALA-PDT for the inactivation of MRSA versus 3T3 fibroblasts. 3T3 cells and MRSA colonies were counted after PDT with 5 mg/mL 5-ALA at three total flux levels. 3T3 cells underwent a loss of viability of 0.27 log_10_-units after exposure to 410-nm light at 50 J/cm^2^ with 5 mg/mL 5-ALA. In contrast, inactivation of MRSA by ∼5 log_10_-units was achieved under similar conditions, indicating that the selectivity for inactivation of MRSA versus 3T3 cells was greater than 5 log_10_-units (Figure 3).

### Accumulation of PpIX in MRSA after systemic administration of 5-ALA

**Figure 4:**
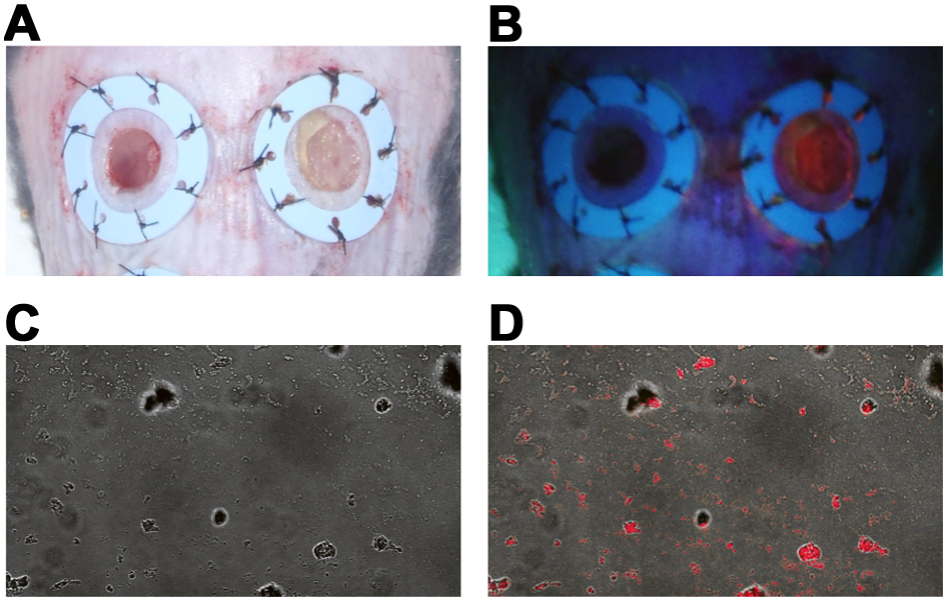
Accumulation of PpIX in MRSA after intraperitoneal injection with 5-ALA. (A) Cutaneous skin ulcers with (right) or without (left) MRSA. (B) Strong red fluorescence was detected in MRSA-infected ulcers 24 h after intraperitoneal injection of 200 mg/kg 5-ALA and irradiation with a Wood’s lamp (right). (C) Transmission image of a smear on a cutaneous ulcer infected with MRSA after intraperitoneal injection of 5-ALA. (D) Confocal laser scanning microscopy of PpIX fluorescence in MRSA. 405-nm diode, ultraviolet light, emission bandwidth 620–650 nm.

The effect of ALA-PDT on MRSA-infected ulcers was studied following systemic administration of 5-ALA *in* *vivo*. Inoculating MRSA (10^10^ CFU/cm^2^) into the skin ulcers of adult male *db/db* mice led to the development of stable infections (Figure 4A). Two days after MRSA inoculation, the wound surfaces were covered with purulent discharge.

We then investigated whether PpIX accumulated in MRSA from skin ulcers after intraperitoneal administration of 5-ALA. Strong red fluorescence was detected at 24 h after 5-ALA injection and irradiation with a Wood’s lamp (Figure 4B). Confocal images of smears from cutaneous ulcers containing MRSA after intraperitoneal injection of 5-ALA suggested the accumulation of PpIX in the bacteria (Figure 4C, D). Thus, PpIX accumulated in MRSA on cutaneous ulcers after systemic administration of 5-ALA.

### Delayed wound healing in the mouse model of MRSA-infected cutaneous ulcers

**Figure 5:**
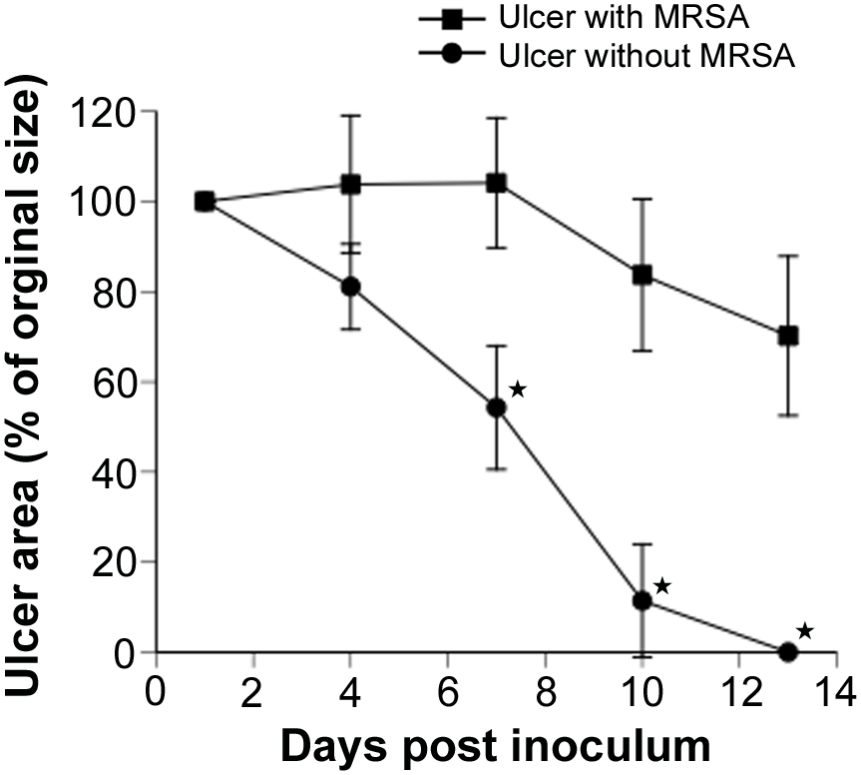
Change in ulcer area over time after MRSA infection. Wound healing over time in MRSA-infected or non-infected cutaneous ulcers. Each point represents the mean percentage of the area of the original wound. Data are expressed as mean ± SEM. *N* = 5 in each group. *P<0.01.

To investigate the effect of ALA-PDT on the healing of MRSA-infected cutaneous ulcers, we compared the speed of epithelialization between ulcers without MRSA infection and ulcers with MRSA infection. Epithelialization of the ulcers was complete by day 13 in all animals without MRSA, as indicated by the ulcer area rate decreasing to 0%. In contrast, the ulcer area rate on day 13 was 70% in animals with MRSA infections (Figure 5). There was a significant delay in the epithelialization of MRSA-infected ulcers after day 7 compared with non-infected ulcers (*P*<0.01).

### Ulcer area after ALA-PDT with various 5-ALA concentrations

**Figure 6:**
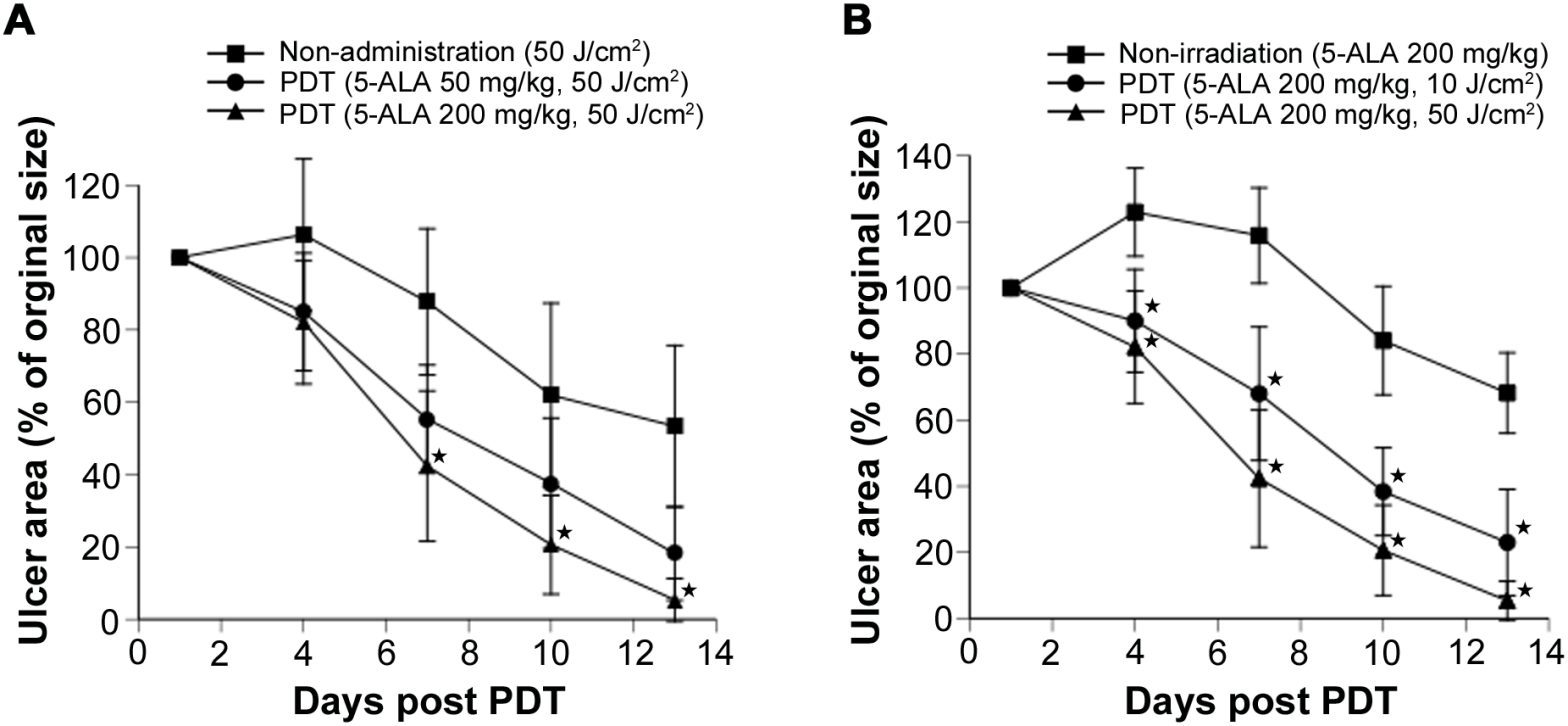
Change in ulcer area over time after PDT. (A) Healing of MRSA-infected cutaneous ulcers treated with 410-nm irradiation at 50 J/cm^2^. (B) Wound healing over time in mice with MRSA-infected cutaneous ulcers treated with 200 mg/kg 5-ALA with or without PDT. Each point represents the mean percentage of the area of the original wound. Data are expressed as mean ± SEM. *N* = 5 in each group. *P<0.01.

Next, we evaluated the changes over time in ulcer area after PDT with various 5-ALA concentrations. PDT was performed with a total flux of 50 J/cm^2^, systemic 5-ALA doses of 0, 50, or 200 mg/kg were applied to mice with MRSA-infected ulcers, and the change in ulcer area was analyzed. When the total flux of 410-nm light was set at 50 J/cm^2^, the ulcer area decreased with increasing 5-ALA doses. After treatment with 200 mg/kg of 5-ALA and LED irradiation at 50 J/cm^2^, the mean ulcer area was 5.4% on day 13 (Figure 6A). This rate was similar to that observed for ulcers without MRSA infection (*P* = 0.17), demonstrating that epithelialization accelerated after PDT.

### Ulcer area after various doses of ALA-PDT

Changes in ulcer area over time were evaluated after PDT with various total flux levels. Specifically, 200 mg/kg of 5-ALA was systemically administered to animals with MRSA-infected ulcers every day, PDT was performed with total flux levels of 0, 10, and 50 J/cm^2^, and the change in ulcer area was monitored over time. We detected no difference in ulcer area rate between treatment with 5-ALA alone and infected ulcers without treatment (*P* = 0.46, data not shown). In contrast, ulcer area declined with increasing irradiation doses (Figure 6B).

### Ulcer area after administration of VCM

**Figure 7:**
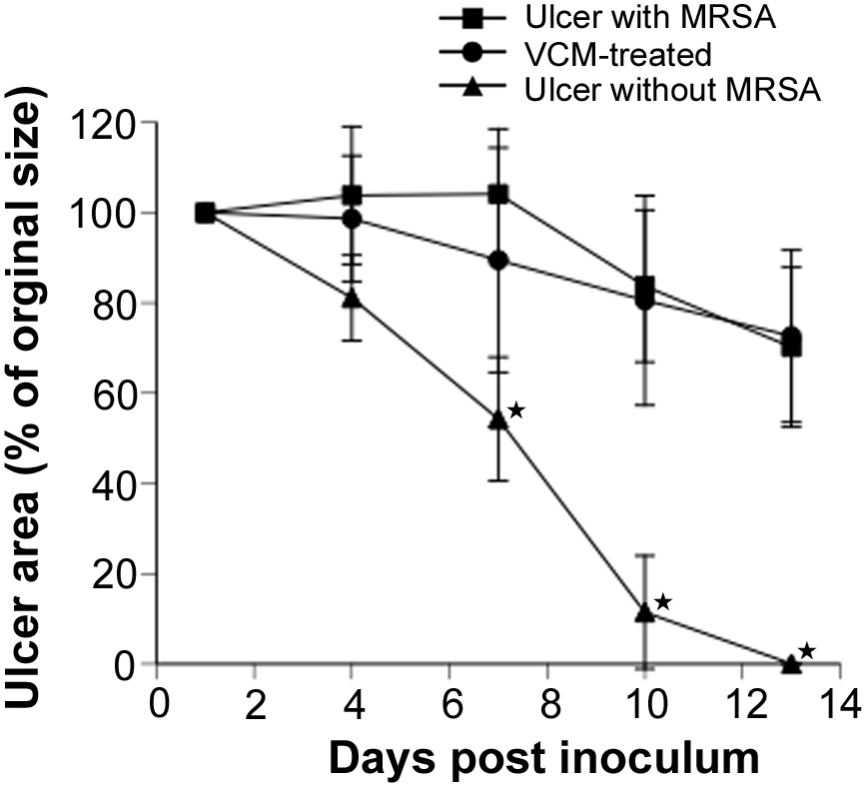
Changes in ulcer area after VCM treatment. Wound healing over time in PDT-treated and VCM-treated mice. Each point represents the mean percentage of the area of the original wound. Data are expressed as mean ± SEM. *N* = 5 in each group.

VCM is a typical antibiotic used for the treatment of MRSA infections. Therefore, we investigated its effect on ulcer area in ulcers without MRSA, ulcers with MRSA, and ulcers with MRSA after treatment with VCM. There was no significant difference in ulcer area rate between mice with MRSA-infected ulcers and the VCM-treated animals on days 4, 7, 10, and 13 (*P*>0.32; Figure 7), suggesting that VCM did not accelerate epithelialization.

### Bacterial counts in ulcer tissue

**Figure 8:**
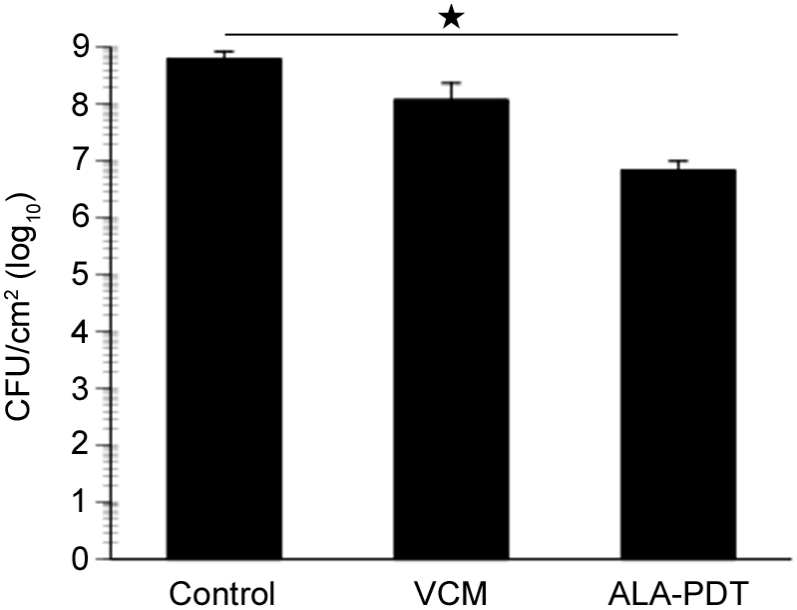
Bacterial counts in wound tissue after PDT. Bacterial cell viability in wound tissue from PDT-treated mice (200 mg/kg 5-ALA and 50 J/cm^2^ irradiation) or VCM-treated mice. Data are expressed as mean ± SEM. *N* = 3 in each group. *P<0.01.

To investigate the bactericidal or bacteriostatic effects of ALA-PDT on MRSA-infected cutaneous ulcers, we counted the viable bacteria in ulcer tissue at 7 days post PDT. PDT-treated mice (200 mg/kg 5-ALA and 50 J/cm^2^ irradiation) exhibited ∼2 log_10_-units of reduction in bacterial viability compared to non-treated mice (Figure 8), suggesting that ALA-PDT accelerates healing via a bactericidal effect against MRSA.

### Gross morphology after ALA-PDT

**Figure 9:**
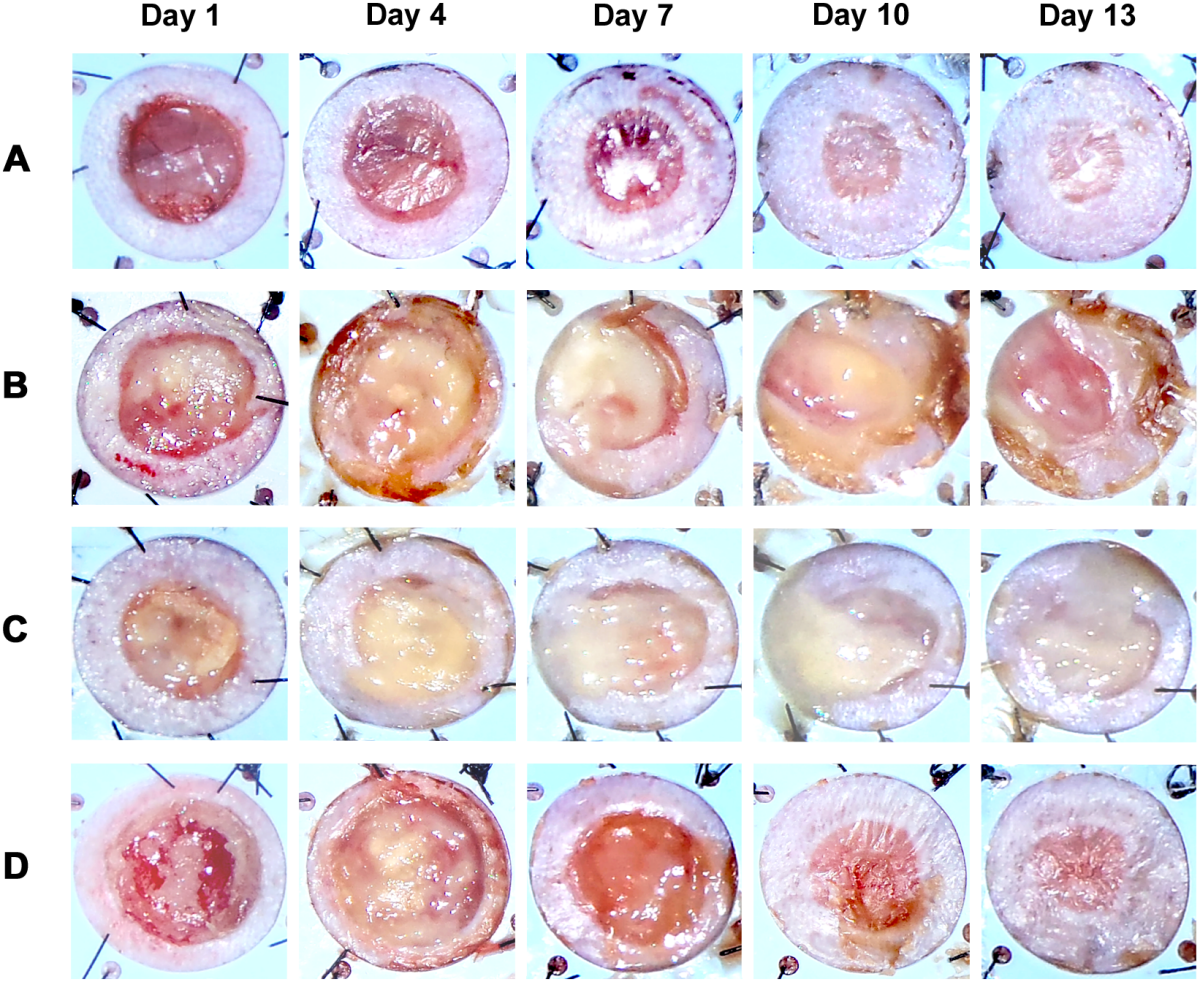
Gross morphology of cutaneous ulcers after PDT. General morphologies of cutaneous ulcers were imaged on days 1, 4, 7, 10, and 13 in (A) C57BL/ksj *db/db* mice without MRSA, (B) C57BL/ksj *db/db* mice with MRSA, (C) VCM-treated mice, and (D) PDT-treated mice (200 mg/kg 5-ALA and 50 J/cm^2^ irradiation).

Finally, we investigated the effects of ALA-PDT on wound healing in mice injected intraperitoneally with 5-ALA; these effects were compared with the effects of VCM exposure as a general treatment for MRSA infection. We macroscopically evaluated purulent discharge, ulcer size, and epithelialization by photo on days 1, 4, 7, 10, and 13 after infection. Ulcer surfaces in VCM-treated mice and non-treated mice were covered with purulent discharge, and epithelialization did not occur by day 13. In contrast, the ulcers of PDT-treated mice (200 mg/kg 5-ALA and 50 J/cm^2^ irradiation) were closed and completely epithelialized 10–13 days after infection. The time to complete wound closure in PDT-treated mice was comparable to that in mice without MRSA infection (Figure 9). These results reveal that PDT with systemic administration of 5-ALA controls MRSA infection and accelerates wound healing.

## Discussion

The present study showed that ALA-PDT with a 410-nm LED exerts an *in-vitro* antibacterial effect on MRSA. Furthermore, our *in-vivo* experiments demonstrated that PpIX accumulates in MRSA on the wound surface after intraperitoneal administration of 5-ALA in mice with MRSA-infected cutaneous ulcers. ALA-PDT accelerated wound healing and decreased bacterial counts on the wound surface, whereas administration of VCM did not accelerate wound healing in this mouse model.

ALA-PDT has been reported to utilize bacterial metabolic pathways; its mechanism of antibacterial activity involves damage to the DNA or cell membrane caused by singlet molecular oxygen or free radicals [18], [19]. To the best of our knowledge, no *in-vivo* study of ALA-PDT has been reported for MRSA. Clinically, an ointment containing 5-ALA is applied to treat skin cancer [13], [23] and acne [24], [25]. However, application of this ointment to extensive wounds or burns with potentially lethal MRSA infections is not practical because more effort is required and because the ointment could be carried away in the exudate. Here, we determined that 5-ALA in the exudate is taken up by MRSA on the wound surface and that PpIX accumulates in MRSA after systemic administration of 5-ALA. Taken together, our results suggest that systemic administration of 5-ALA combined with use of an LED for wide irradiation could be used to treat extensive wounds.

Blue light is known to exhibit antimicrobial activity, which has been reported to be due to the activation of endogenous microbial porphyrins [26]. Previously, irradiation with blue light at ∼50 J/cm^2^ decreased the bacterial count of MRSA by nearly 1 log_10_-unit [27]–[29], but no previous investigation has employed a 410-nm LED for ALA-PDT targeting MRSA. We achieved favorable antibacterial activity against MRSA via ALA-PDT with a 410-nm LED. PpIX has a large absorption coefficient at 410 nm, where the absorbance is ∼30-fold higher than at 635 nm (the maximal excitation efficiency [14]). Thus, antimicrobial activity can be expected when PDT is performed with a 410-nm LED. When an *in-vitro* study of ALA-PDT for MRSA was carried out with a halogen lamp, a decrease of 5 log_10_-units followed irradiation at 120 J/cm^2^ [30]. In the present investigation, a similar effect was obtained after irradiation at 50 J/cm^2^, indicating that a wavelength of 410 nm is more effective for PDT than other wavelengths. Generally, a wavelength of 630 nm is employed for PDT of bacteria or tumors due to the greater depth of penetration [31], [32]. However, a wavelength of 410 nm efficiently excites PpIX at superficial locations, and ALA-PDT using blue light has already been employed to treat acne [33]. A wavelength of 410 nm is considered to be useful for treating infected cutaneous ulcers and burns or other superficial MRSA infections. In addition, LED light sources have several advantages over lasers; they are cheaper and can deliver irradiation over a wide area.

In this study, a decrease of 5 log_10_-units was observed *in* *vitro*, but the *in* *vivo* decrease was only 2 log_10_-units. This difference may have occurred because the 5-ALA concentration at the ulcer surface after systemic administration was lower than the concentration employed for the *in-vitro* experiment.

The results of this study indicate that ALA-PDT accelerates wound healing in mice with MRSA-infected skin ulcers. Although wound healing can occur when the bacterial count in granulation tissue is <10^5^ CFU/g [34], the bacterial count was not reduced to such a level in the present study. PDT has been reported to exert an immunostimulatory effect [31], [35], [36] and to promote the accumulation of neutrophils, preventing infection [37],[38]. Clinically, topical ALA-PDT has been employed for the photorejuvenation of facial skin by stimulating cellular differentiation and proliferation [39]. Thus, it is possible that not only the direct antimicrobial effect of ALA-PDT, but also its influence on the immune system, was involved in the acceleration of wound healing in the present study.

Several reports used various photosensitizers for *in-vitro* PDT for MRSA; 4–6 log_10_-unit decreases in bacterial count were reported with methylene blue [40], 3–4.47 log_10_-unit decreases occurred with toluidine blue O [41], [42], a decrease of 5.48 log_10_-units followed treatment with aluminum disulphonated phthalocyanine [43], a decrease of 5.4 log_10_-units was observed with cationic porphyrin photosensitizer XF73 [44], >6 log_10_-unit decreases were associated with hypericin exposure [45], and a decrease of 4 log_10_-units was detected with photofrin [46]. Therefore, the antibacterial effect of the ALA-PDT employed in the present investigation was similar to that of previous reports. The *in-vivo* kinetics of 5-ALA, which has attracted attention as a third-generation photosensitizer, have been studied in detail, and this agent has already been put to practical use for the treatment and intra-operative diagnosis of cancer [47]. Since 5-ALA and PpIX are endogenous substances, PDT with 5-ALA has the advantage of rapid metabolism/elimination compared to other photosensitizers. PDT with 5-ALA also carries little risk of phototoxicity.

Due to its mechanism of action, ALA-PDT cannot lead to bacterial resistance, and can be used concomitantly with antibiotics or other treatments against MRSA. Thus, ALA-PDT may become a new treatment option for MRSA infections. Further studies are needed to assess its influence on the immune system and its effect on bacterial flora after MRSA is removed from the bacterial community in the skin.
